# Parallel Key Frame Extraction for Surveillance Video Service in a Smart City

**DOI:** 10.1371/journal.pone.0135694

**Published:** 2015-08-18

**Authors:** Ran Zheng, Chuanwei Yao, Hai Jin, Lei Zhu, Qin Zhang, Wei Deng

**Affiliations:** Services Computing Technology and System Lab, Cluster and Grid Computing Lab, School of Computer Science and Technology, Huazhong University of Science and Technology, Wuhan, Hubei, China; Shenzhen Institutes of Advanced Technology, CHINA

## Abstract

*Surveillance video service* (SVS) is one of the most important services provided in a smart city. It is very important for the utilization of SVS to provide design efficient surveillance video analysis techniques. Key frame extraction is a simple yet effective technique to achieve this goal. In surveillance video applications, key frames are typically used to summarize important video content. It is very important and essential to extract key frames accurately and efficiently. A novel approach is proposed to extract key frames from traffic surveillance videos based on GPU (*graphics processing units*) to ensure high efficiency and accuracy. For the determination of key frames, motion is a more salient feature in presenting actions or events, especially in surveillance videos. The motion feature is extracted in GPU to reduce running time. It is also smoothed to reduce noise, and the frames with local maxima of motion information are selected as the final key frames. The experimental results show that this approach can extract key frames more accurately and efficiently compared with several other methods.

## Introduction

With the development of various intelligent techniques, smart cities have attracted much attention in recent literature from both researchers and practitioners because of their great application values for facilitating social activities. According to the requirements of a smart city, city management system should provide many convenient and intelligent services to the public.

*Surveillance video service* (SVS) is one of the most important services in a smart city. From massive surveillance videos, valuable features, such as trajectories or visual appearances of moving objects, are captured and form the basis for intelligent traffic surveillance and public security. Therefore, it is important to design intelligent video analysis techniques to efficiently mine this latent and valuable information. One of the most important strategies to adopt is leveraging effective machine learning techniques to analyze heterogeneous information involved in surveillance videos.

Surveillance videos are comprised of a large amount of frames (images). Any video analysis process is conducted on the original raw frames. It is common that subsequent frames in videos generally have small differences, and there is redundant information among them. Therefore, video analysis performed on these raw frames is a time-consuming process. To accelerate this process, however, researchers have proposed the technique of key frame extraction to represent the information of the entire video with a compact frame set.

In videos, key frames indicate a limited frame subset to represent the major contents of video sequences [1]. The use of key frames can greatly reduce the amount of video information, achieve video data transmission at a low bit rate channel, reduce the physical memory space, and provide convenience for the users to view the main content of the video [2]. The technology of key frame extraction has important significance in SVS, and it has attracted extensive attention from other various fields. In this case, the accuracy and efficiency of key frame extraction has great impact on the subsequent video analysis.

Although researchers have proposed many approaches for key frame extraction, they have generally devoted their efforts to improving the accuracy of key frame extraction and failed to consider the execution efficiency. In this paper, an accurate and efficient key frame extraction approach is proposed by comprehensively considering both algorithm design and implementation. For algorithm design, a motion feature-based approach is chosen for the determination of key frame selection. For algorithm implementation, a computational process of the designed sequential algorithm is parallelized on modern *graphics processing units* (GPU) to improve the extraction efficiency, and several effective GPU optimization strategies are proposed. Motion feature-based key frame extraction can effectively capture visual variations of consecutive frames, while GPU-based parallel implementation can fully leverage rich resources and the powerful parallel processing capability of the underlying hardware to efficiently accelerate the designed sequential algorithm.

The remainder of this paper is organized as follows. Section 2 introduces the related work. Section 3 presents the implementation and optimization details of the proposed approach. The GPU-based parallelization of motion information computation is given in Section 4. The experimental results are presented in Section 5. Section 6 concludes the paper and indicates some possible directions for further research.

## Related Work

### 2.1. Sequential Key Frame Extraction

Current techniques for extracting key frames can be classified into four categories according to various measurements [3, 4].

The comparison-based method sequentially compares each frame with the previously extracted key frame with feature differences. A frame can be selected as the key frame only if its features significantly differ from that of the previously extracted key frame. This method is very simple and easy to implement. However, the extracted key frames represent only local properties and may generate redundant information.

The reference frame-based method [5, 6] adopts certain methods to generate a virtual reference frame, and then each frame is compared with the reference frame. Finally, the key frame is chosen based on the comparison results. This method is also easy to understand and implement, but the accuracy of the method is largely dependent on the selection of the reference frame.

The clustering-based method [7] divides a video sequence into clusters, and each cluster is considerably different from the other clusters, while the frames within a cluster have high similarity. The frames closest to the cluster centers are selected as key frames. However, the extracted key frames may not preserve the sequential information of the video sequence.

The objects/events-based method [1, 8] considers that the key frames should represent changes in motion or specific events. These algorithms detect the information about objects or events for each frame and extract key frames based on this information. The extracted key frames can reflect the motion pattern of objects or events, which is semantically important. However, the detection of objects or events relies strongly on the specified heuristic rules according to the application. Additionally, these algorithms are generally computationally expensive.

### 2.2. GPU and CUDA

In recent years, there has been a trend to use GPUs as accelerators for general-purpose computing [9]. To enable flexible programmable graphics, NVIDIA developed a hardware/software architecture called the *Compute Unified Device Architecture* (CUDA) [10].

As the process of key frame extraction is data-intensive and time-consuming, some studies have ported sequential processes to the GPU to reduce processing time. Kehoe et al. [11] implemented a representative key frame selection on a GPU that first detects the shot boundaries. Then, it calculates the difference between each frame and every other frame. Finally, the frame with the lowest average distance between itself and every other frame is selected as the key frame.

## Proposed Method

### 3.1. Motion Information

Traffic surveillance videos generally have static backgrounds, and not much valuable information is contained in them. The most salient and attractive objects are vehicles and pedestrians, and the key frame should contain as many of these objects as possible with a good viewing angle. More specifically, a summarization of a traffic surveillance video by key frames should contain all objects in a minimum number of frames.

Motivated by the above premises and reasons, motion information is selected to determine key frame extraction. The *motion information* (MI) of a frame is defined as the number of foreground pixels. Let *W* and *H* be the width and height of a frame, respectively, and *P*_*(i*, *j)*_ *(k)* be the binary value of a pixel in the *i* ^*th*^ row and *j* ^*th*^ column within the *k* ^*th*^ frame. *P*_*(i*, *j)*_ *(k)* is 1 if the pixel is a foreground pixel; otherwise, it is 0. For the *k* ^*th*^ frame, *MI(k)* is calculated as follows:

$$MI(k)=\sum^{\text{​}}P_{\left(i,j\right)}(k)\;0<=i<W,0<=j<H$$ (1)

Motion information can generally represent the amount of motion within a video. A frame can be selected as the key frame if its motion information is larger than that of its neighboring frames.

### 3.2. Process Flow

A novel approach is proposed to extract key frames from traffic surveillance videos based on GPU. The process flow of this approach is demonstrated in S1 Fig.

First, the background is extracted from the source video with an adaptive Gaussian mixture models method [12]. Based on the background, the foreground image of each frame is obtained using the background subtraction method. Second, the motion information is computed on the GPU to reduce the processing time, and a smooth process is implemented on the motion information sequence to reduce the interference of noise and false foreground pixels. Finally, the local maxima of the motion information sequence are computed, and the frames with the local maxima of motion information are chosen as the final key frames.

### 3.3. Smooth Motion Information

During the foreground extraction process, some noise is inevitably generated and some background pixels may be detected as foreground pixels. These noises and fake foreground pixels may have a negative effect on the key frame selection accuracy. Thus, it is necessary to smooth the motion information curve to reduce the noise. For the *k* ^*th*^ frame, the smoothing process is calculated as follows:

$$MI(k)=\frac{1}{2T1}\;\sum_{i=-T1}^{T1}MI(k+i)$$ (2)

*T1* is the size of the smoothed window, and the experimental value of *T1* is 10. The motion information of every frame is the mean value of the summation of its motion information of the neighboring frames within the window *T1*.

### 3.4. Local Maxima Extraction and Key Frame Selection

For surveillance video applications, the key frame should contain as many moving objects as possible with the most compact frame set. From this viewpoint, the value of motion information in the key frames should be the local maxima over the video sequence. This is similar to a metric in optical flow [13] that is used to select the key frame with various motion-based methods.

Based on the smoothed motion information obtained from the previous step, the local maxima of the motion information sequence are extracted to select the key frames. For the *i* ^*th*^ frame, it is determined whether *MI(i)* is the largest value of the sequence [*MI(i–T2)*, *MI(i–T2–1)* …. *MI(i + T2–1)*, *MI(i + T2)*]. If *MI(i)* is the largest value, the *i* ^*th*^ frame is marked as the key frame and the *(i+T2)* ^*th*^ frame is processed; otherwise, the next frame is processed. *T2* is the size of sliding window, and its value can be changed based on the requirements. If more key frames are needed, *T2* can be set to an even smaller value. The experimental value of *T2* is 100.

## Motion Information Computation on GPU

While the foreground information is generated, the motion information for each frame is computed on the GPU. There are generally two ways of mapping from frame data to the GPU logic-computing module. One method is Frame-Per-Thread, which allocates one thread to process one frame. The other is Frame-Per-Block, which allocates one block to process one frame.

The Frame-Per-Thread method is very simple and easy to implement, but it cannot make full use of GPU resources because the number of threads executed concurrently is limited by the size of the GPU memory space. In this case, the workload of each thread is huge. For the Frame-Per-Block method, one block (generally containing 256 or 512 threads) processes one frame, and the workload of each thread is lighter. However, the final reduction and summation will longer processing time because the processing contains a branch statement, which can lead to lower efficiency. Considering the workload of each thread and the efficiency of the reduction and summation operations, the Frame-Per-Warp method is adopted in this approach, which allocates one warp to process one frame.

A warp refers to a cluster of 32 threads that are “woven together,” and they are executed in lockstep. At each line of the program, each thread in a warp executes the same instruction on different data with high efficiency. Within the Frame-Per-Warp method, the workload of each thread is moderate. The reduction and summation operation within a warp is faster than the operation within a block. S2 Fig shows the differences between the three methods.

The details of the Frame-Per- method are described as follows:

- Shared memory allocation and initialization. The shared memory (a memory area within each block with high access efficiency) is allocated and initialized to 0. The shared memory stores temporary motion information results for each frame with low latency and high efficiency.
- Execution of the summation for each thread. Due to global memory access efficiency, the threads within a warp continuously access the frame data (in the global memory). The *t* ^*th*^ thread accesses the *t* ^*th*^, *(t + 32)* ^*th*^, (t + 32*2) ^*th*^ … pixel data, and the *(t + 1)* ^*th*^ thread accesses the *(t + 1)* ^*th*^, *(t + 1 + 32)* ^*th*^, (t + 1 + 32*2) ^*th*^ … pixel data. Each thread adds motion data to the shared memory. For the *t* ^*th*^ (0 ≤ *t* ≤ 31) thread within the *k* ^*th*^ warp, *MI(k*, *t)* is calculated:
  $$MI(k,t)=\text{​}\text{​}\text{​}\text{​}\;\sum^{\text{​}}P_{\left(\;(t+32\times n)/W,\;(t+32\times n)\%W\;\right)}\;(k)\;\;\;0<=n<\frac{W\times H}{32}$$ (3)
- Parallel reduction and summation. The final motion results of each frame are summarized within a warp. It is important to parallelize the reduction and summation, which can have a great effect on the GPU computation performance. Shared memory bank conflict and highly divergent warps are two fatal problems that seriously affect kernel efficiency in parallel reduction. In [14], a sequential addressing method is adopted to avoid divergent branching and bank conflict [15]. The same method is used here to process the reduction and summation, as illustrated in S3 Fig. The sequential order of the activated threads can avoid the warps to be discrete and conflicted. In each iteration, the motion results of the back threads are summarized into the front threads. After several rounds of iterated circulation, the *0* ^*th*^ thread within each warp will keep the final motion information of each frame. Thus, the *0* ^*th*^ thread within each warp transfers the final summation results back to the global memory.

## Experimental Results

### 5.1. Experimental Environment

Motion information computation is implemented on an NVIDIA GeForce GTX 295 GPU and K20. Experiments are conducted on the publicly available dataset from [16], as well as the campus road surveillance video from the university (the video is provided by the Security Department of HUST for scientific research, which is also publicly available and freely open-use information) [17].

The first testing dataset from [16] consists of various real-world videos in six semantic categories. It provides the ground truth of foreground video for each video. These foreground videos are used to verify the accuracy of the method. Two videos are chosen as the test video: “Highway” from the changed detection dataset and “Campus-road” from the university.

### 5.2. Accuracy Evaluation

For the accuracy evaluation, the method is compared with the method of sequential comparison of *color* (H) [5], *color spatial distribution histograms* (CSH) [18], *localized foreground entropy* (LFE) [19], and *twin-comparison* (TC) [20]. As there are no benchmarking or ground truth results for key-frame extraction algorithms thus far, so the ground truths of the “Highway” and “Campus-road” videos are manually labelled by a natural principle that the key frame should contain as many objects as possible.

The recall and precision for the entire video sequence are calculated according to the definitions in [12], where recall is the fraction of relevant information units (set *Ri*) that has been retrieved, and precision is the fraction of the retrieved information units (set *Ai*) that is relevant Eq (4):

$$recall=\frac{\left|Ri\;\cap^{\text{​}}\;Ai\right|}{\left|Ri\right|},\;precision=\;\frac{\left|\;Ri\;\cap^{\text{​}}\;Ai\right|}{\left|Ai\right|}$$ (4)

S1 Table summarizes the results of recall and precision (shortened to R and P, respectively) for these five different methods.

To evaluate the foreground extraction accuracy, this method is validated under two conditions: one uses the *ground truth* (GT) method and the other uses the foreground extracted by the adaptive GMM. There is no ground truth foreground for “Campus-road” video; therefore, the results for GT are not listed in S1 Table, where H stands for the histogram method and TC represents the twin-comparison method.

It is clear from S1 Table that the proposed method and the LFE method have better accuracy in recall as well as precision because both methods focus on motion features. The foreground video is not the ground truth, thus GMM achieves poorer results for recall and precision compared with GT.

### 5.3. Extraction Speed Evaluation

For evaluation of the extraction speed, the proposed method is implemented on a CPU and GPU. The Frame-Per-Thread, Frame-Per-Warp, and Frame-Per-Block methods are implemented for performance comparisons on the GPU.

S4 Fig presents the speed comparison of the three different GPU implementations over CPU implementation. It can be easily observed from S4 Fig that the Frame-Per-Warp implementation is approximately 9.5 ~ 10x faster compared with CPU implementation on GTX 295, and 8.5 ~ 12.5x faster compared with CPU implementation on K20. For the GPU implementation, the Frame-Per-Warp method is faster than the other two GPU methods because in the Frame-Per-Warp method, the workload of each thread is moderate and the parallel summation and reduction can be handled with higher efficiency.

The proposed method is also compared with three other methods: histogram, LFE, and twin-comparison. The CSH method takes too much time; therefore, its time results are not presented. S5 Fig illustrates the comparison of the speed improvement obtained by the GPU implementation compared to the other three methods. It is shown that, on GTX 295, the GPU implementation can achieve approximately 11x more speed compared with the twin-comparison method, 10x more speed compared with the LFE method, and 8.5 ~ 9.2x more speed compared with the histogram method. On the K20, the GPU implementation can achieve 9.5 ~ 13.5x more speed compared with the twin-comparison method, 8.8 ~ 12.2x more speed compared with the LFE method, and 7.8 ~ 10x more speed compared with the histogram method.

## Conclusion and Future Work

In this paper, a novel approach is proposed to extract key frames from traffic surveillance videos based on GPU processing. For accuracy, motion information is smoothed, and the frames with local maximum values of motion information are selected as key frames. Compared with several other methods, the experimental results show that this method performs better in recall and precision. To improve the performance, this method is implemented on a GPU and the Frame-Per-Warp method is developed to obtain the optimization for GPU implementation. In future, other features (such as colors, edges, and events) will be combined with motion information for key frame selection to further improve the precision. Furthermore, the algorithm will be run on a GPU cluster to be increase the process speed and obtain higher performance.

## Supporting Information

S1 Fig Process flow of the proposed approach. (TIF)

S2 Fig Three different kinds of mapping from frame data to GPU. (TIF)

S3 Fig Parallel reduction and summation with no conflict. (TIF)

S4 Fig Increase in speed of three different GPU implementations compared to the CPU implementation. (TIF)

S5 Fig Increase in speed of GPU implementation compared to the other three methods. (TIF)

S1 Table The comparison of recall and precision for five different methods. (XLS)
