# Supplementary figures and images for: Parallel Key Frame Extraction for Surveillance Video Service in a Smart City
Source: PLoS One. 2015 Aug 18;10(8):e0135694. doi: 10.1371/journal.pone.0135694 (PMC4540463; doi:10.1371/journal.pone.0135694)

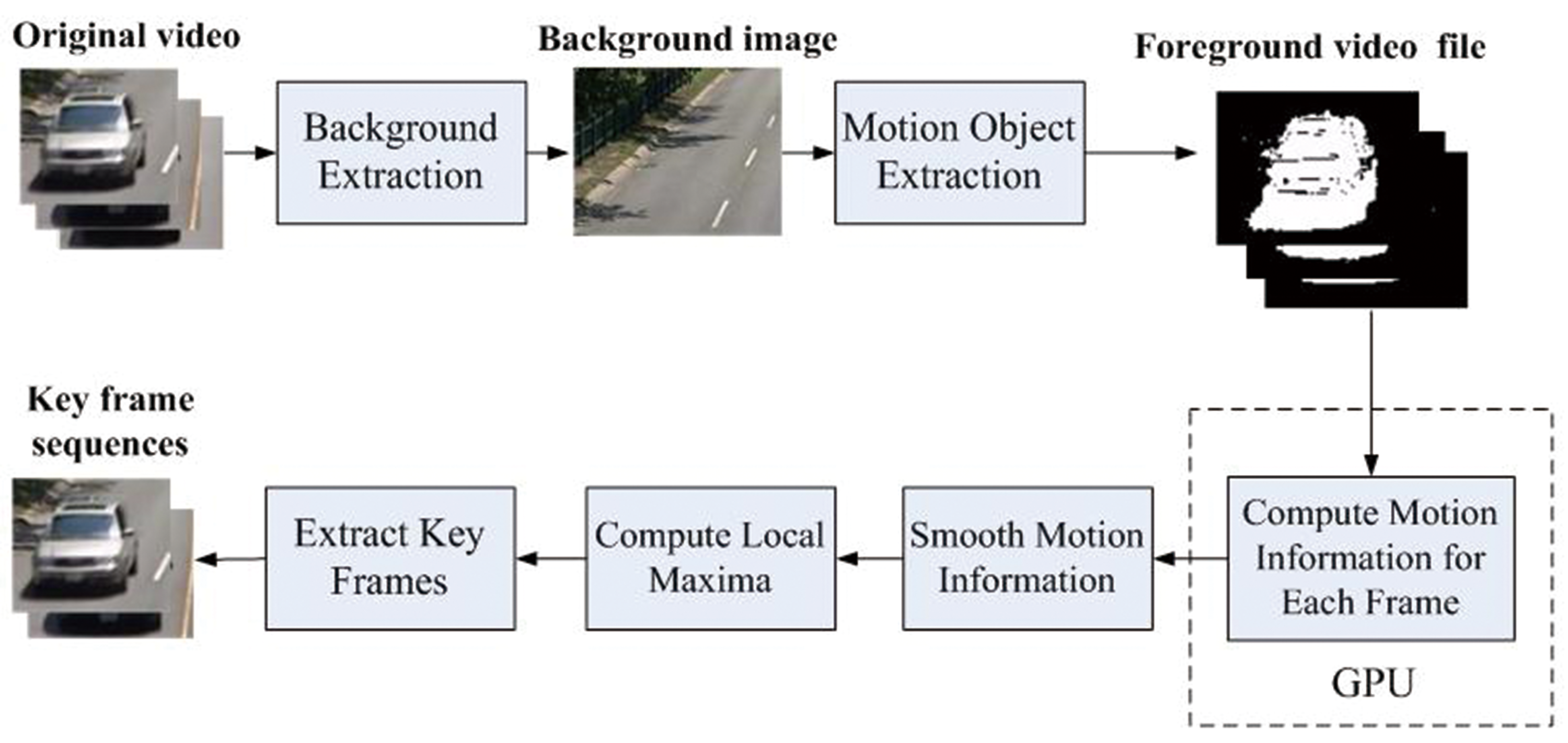

Supplement: S1 Fig — (TIF) [file pone.0135694.s001.tif]

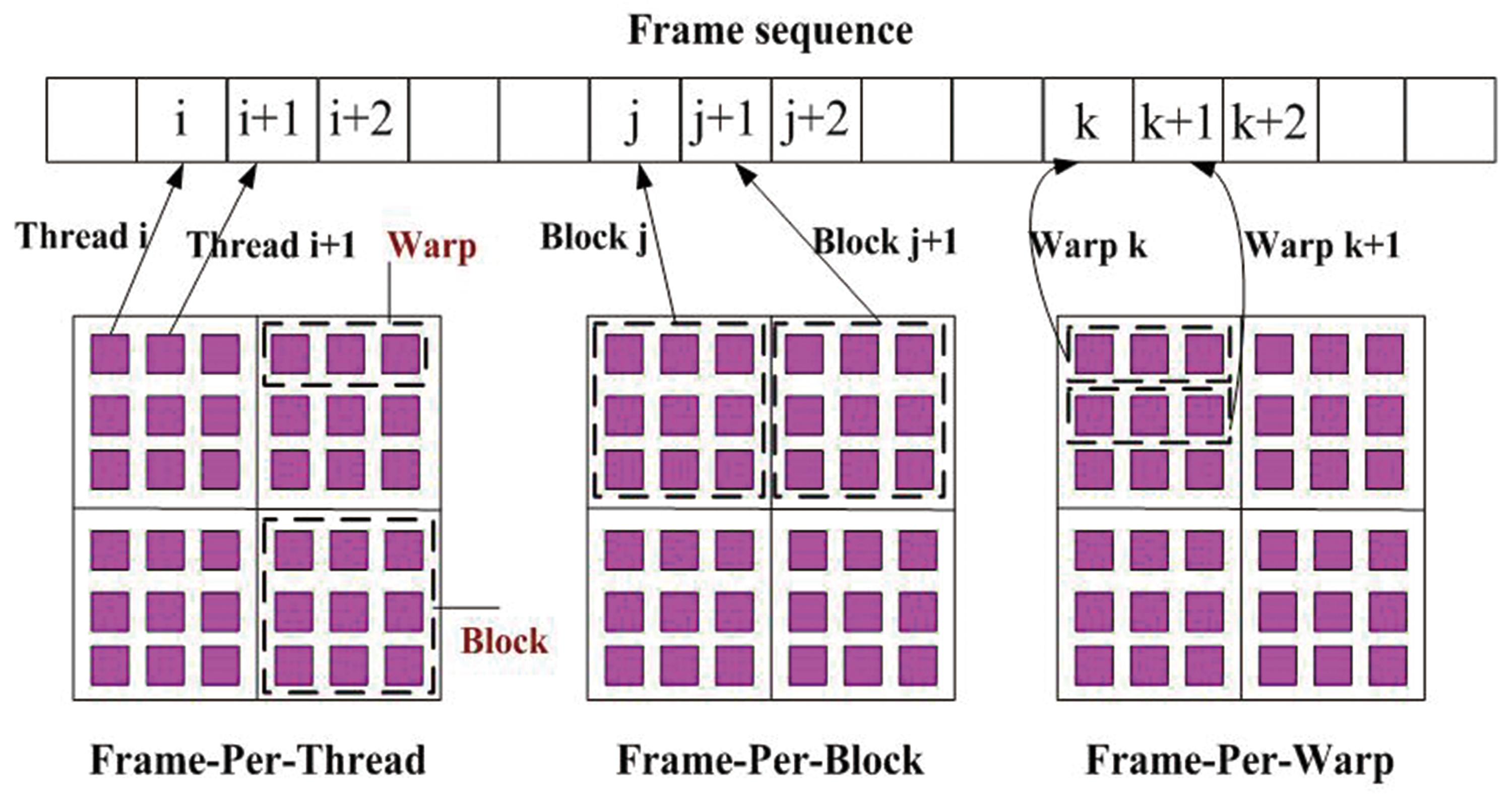

Supplement: S2 Fig — (TIF) [file pone.0135694.s002.tif]

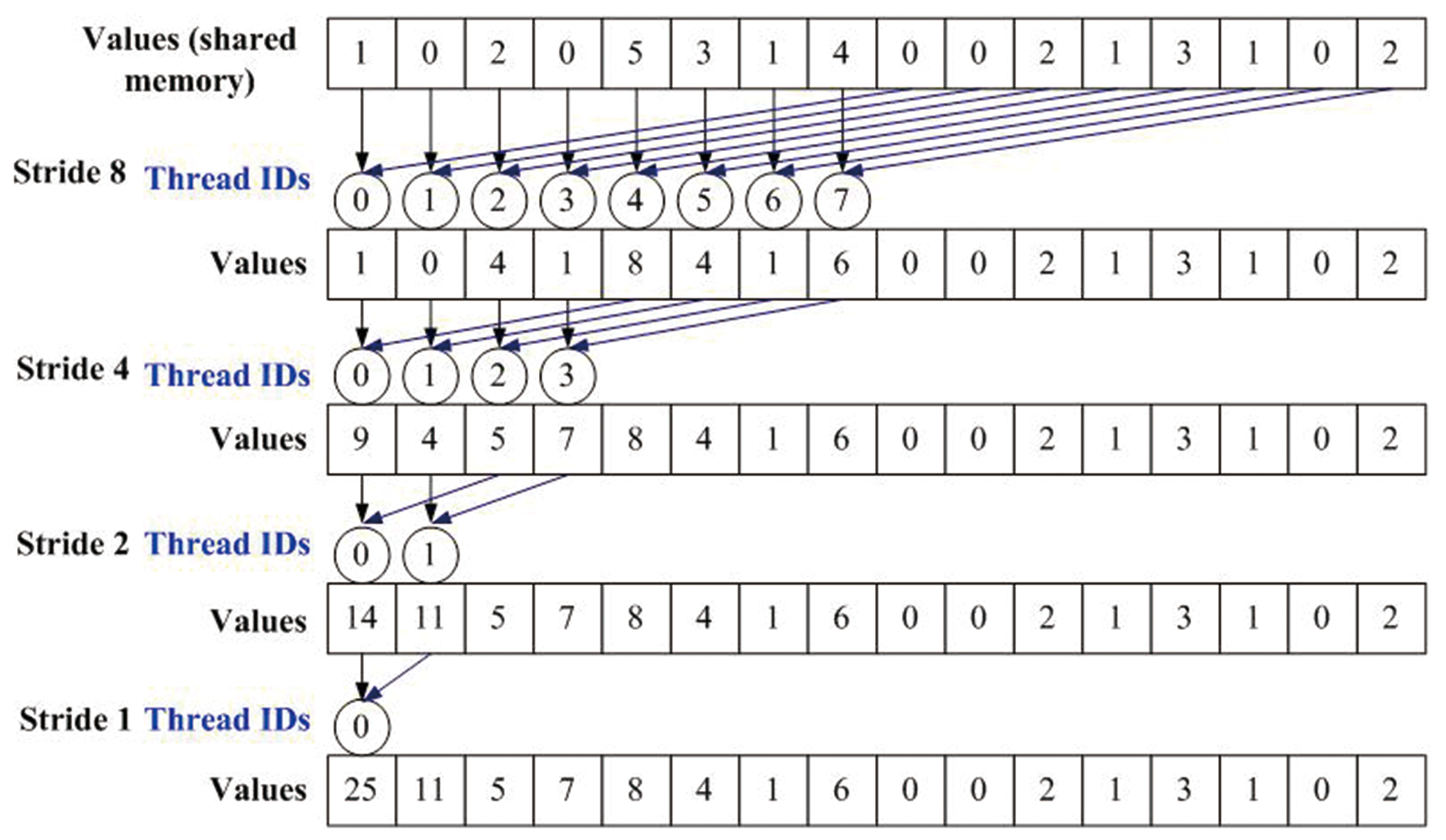

Supplement: S3 Fig — (TIF) [file pone.0135694.s003.tif]

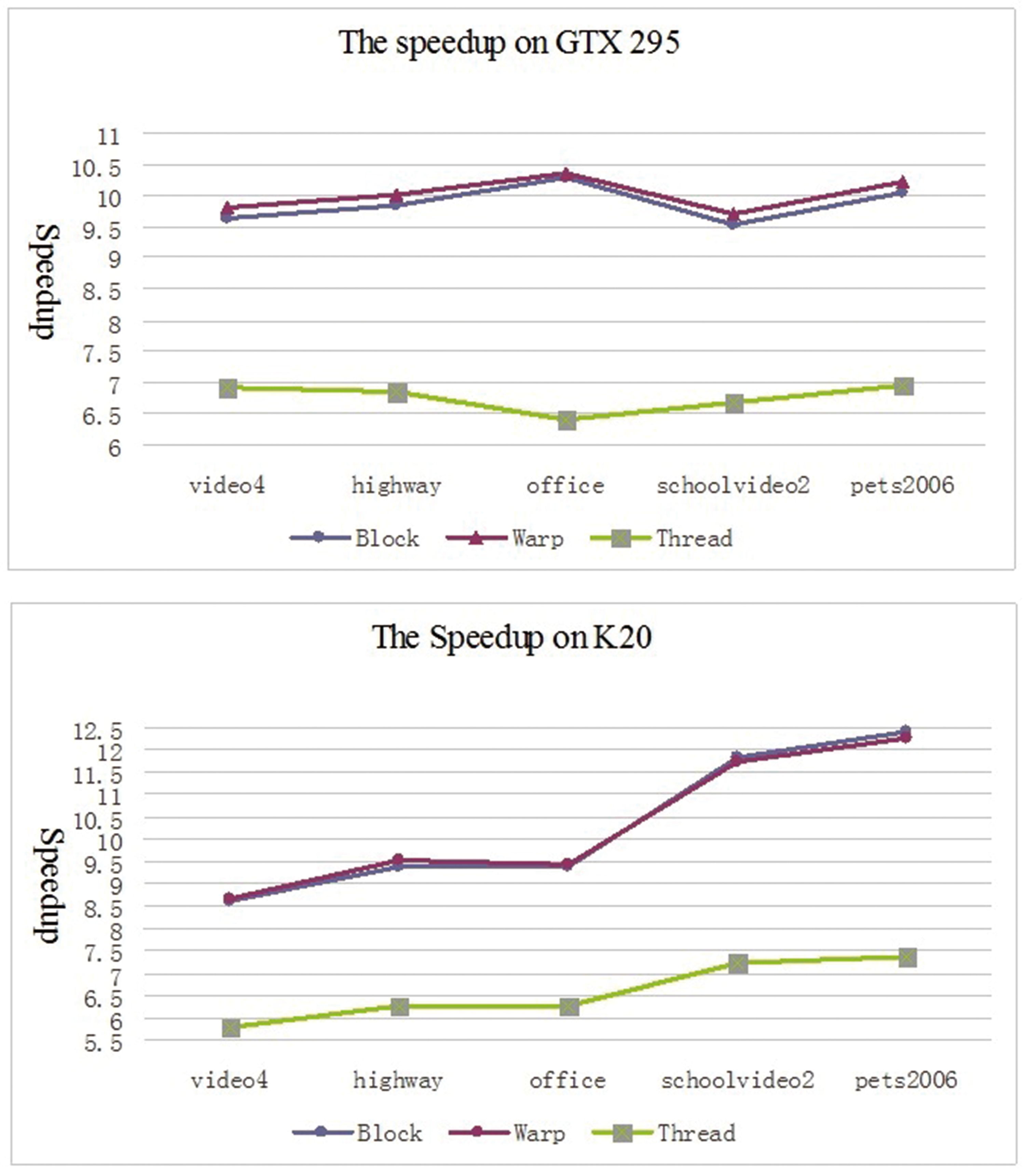

Supplement: S4 Fig — (TIF) [file pone.0135694.s004.tif]

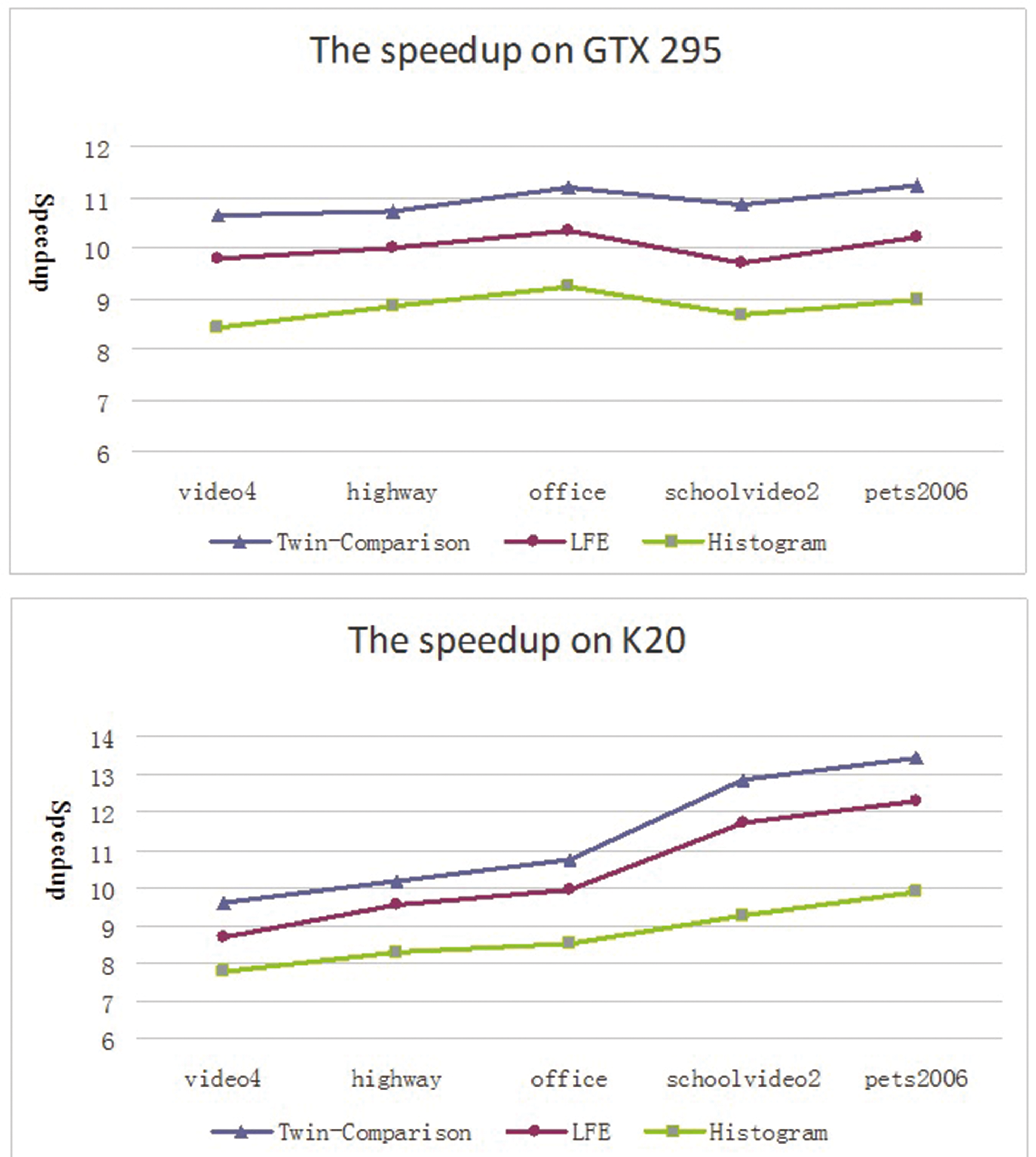

Supplement: S5 Fig — (TIF) [file pone.0135694.s005.tif]
